# Steel surface defect detection method based on improved YOLOv9

**DOI:** 10.1038/s41598-025-10647-1

**Published:** 2025-07-11

**Authors:** Cong Chen, Hoileong Lee, Ming Chen

**Affiliations:** 1 School of Marine Information Engineering, Hainan Tropical Ocean University, Sanya, 572022 China; 2 Faculty of Electronic Engineering & Technology, Universiti Malaysia Perlis, Arau, Perlis 02600 Malaysia; 3 School of Information and Intelligent Engineering, University of Sanya, Sanya, 572022 China

**Keywords:** YOLOv9, Steel surface defect detection, DSConv module, C3 module, BiFPN module, DySample upsampling operator, Materials science, Mathematics and computing

## Abstract

With the development of industrial automation and intelligent manufacturing, steel surface defect detection has become a critical step in ensuring product quality and production efficiency. However, the diverse types and significant size variations of defects on steel surfaces pose great challenges. Among these defects, small-sized defects are characterized by their subtle appearance on the surface, making them difficult to distinguish from the background. This often results in high false detection and missed detection rates during the inspection process. To address this issue, this paper proposes an improved steel surface defect detection algorithm based on YOLOv9. First, introducing Depthwise Separable Convolution (DSConv) can effectively reduce the computational complexity of the model, thereby enhancing its operational efficiency. Second, the C3 module is incorporated to effectively fuse feature maps from different levels, enhancing the model’s ability to detect multi-scale targets. To improve the recognition accuracy of small targets, a bidirectional feature pyramid network (BiFPN) is integrated, enabling the model to capture small target features more precisely. Additionally, the DySample upsampling operator is employed to address the issue of detail loss in traditional upsampling methods, enhancing the model’s sensitivity and localization accuracy for small targets. Experimental results demonstrate that the improved model achieves a mean average precision (mAP) of 78.2% and an accuracy of 82.5%, representing increases of 1.8% and 7.4%, respectively, compared to the baseline model. Moreover, the number of model parameters is reduced by 8.9%. The findings of this study hold significant practical value for improving the quality and efficiency of industrial products in the field of steel surface defect detection.

## Introduction

With the rapid development of Industry 4.0 and intelligent manufacturing, the demand for quality inspection of industrial materials such as steel has been steadily increasing. In particular, the automated detection of surface defects has become a critical step in ensuring product quality and production efficiency^1^. However, due to the complexity of production processes and the influence of environmental factors, various surface defects frequently occur during the production and transportation of steel. Among these, small-sized defects are especially challenging, as their dimensions are often extremely small, typically in the millimeter or even micrometer range, and their surface morphology is irregular, making them difficult to perceive through conventional visual or tactile methods. For example, the length and width of microcracks may be less than 1 mm, while the diameter of pores or bubbles is often only a few tens of micrometers. These tiny defects become even more concealed when covered by coatings or rust. Traditional manual inspection methods are not only inefficient^2^ but also prone to human error, resulting in inconsistent and inaccurate detection outcomes. Consequently, automated detection technologies based on deep learning have emerged as a research hotspot. Among them, the YOLO (You Only Look Once)^3–13^ series of models have been widely applied in the field of object detection due to their advantages in fast detection and high accuracy. However, despite the remarkable success of YOLO models in general object detection tasks, significant challenges remain in applying them to the specific domain of steel surface defect detection, particularly in handling small object detection.

To overcome these challenges, researchers in recent years have proposed various improvement methods. Song et al.^14^ proposed an improved YOLOv8-based steel surface defect detection algorithm that incorporates deformable convolutional networks (DCN) and the BiFormer attention mechanism to enhance the detection of complex textures and irregularly shaped defects. However, the model’s performance in handling complex backgrounds and small targets still requires improvement. Yang et al.^15^ proposed an improved YOLOv8s model that combines CNN and Transformer architectures to optimize feature extraction and reduce computational complexity, thereby enhancing the detection performance of steel surface defects. Nonetheless, the model still faces challenges in detecting small targets in complex backgrounds. Wang et al.^16^ introduced a lightweight steel surface defect detection model named DAssd-Net, which enhances feature learning and detection capabilities through multi-branch dilated convolution aggregation and a multi-domain perception detection head. While the model achieved success in reducing computational costs and model size, its accuracy decreased when dealing with complex defects. Liu et al.^17^ proposed an improved YOLOv4-based method for steel surface defect detection, which replaces traditional convolution operations with depthwise separable convolution (DSConv) to reduce model parameters and computational complexity while enhancing feature extraction capabilities and model lightweighting. Although it is suitable for environments with limited computational resources, further accuracy improvements are needed. Zhou et al.^18^ proposed a hybrid attention network that combines multi-scale feature fusion with lightweight design to improve steel surface defect detection performance. While this method significantly improved detection speed, its performance was still suboptimal for detecting multiple types of defects. Wei et al.^19^ introduced an improved method based on feature pyramid networks (FPN), which enhances detection accuracy for large-scale targets through multi-scale feature fusion but still struggles with detecting small targets in complex backgrounds. Xie et al.^20^ proposed an improved YOLO-based detection method that utilizes multi-layer feature fusion to enhance the accuracy of steel surface defect detection. However, the method’s complex structure leads to high computational costs, limiting its applicability in real-time scenarios. Xu et al.^21^ developed a multi-layer feature fusion technique that integrates multi-scale features to improve the accuracy of steel surface defect detection. While this method showed improvements in accuracy, its performance may decline in complex backgrounds or noisy environments. Nath et al.^22^ proposed the NSLNet model, which optimizes steel surface defect classification performance on small datasets through data augmentation and attention mechanisms. Although this method improved detection accuracy, its relatively complex structure led to increased computational costs, restricting its application in resource-constrained environments. Yang et al.^23^ proposed an improved YOLOv8s model that optimized the feature extraction module and introduced the DySample upsampling strategy to enhance detection accuracy and efficiency. However, the model performed poorly in complex scenarios, resulting in an imperfect balance between detection speed and accuracy.

In summary, although existing methods have achieved some progress in steel surface defect detection, numerous technical challenges remain, particularly in detecting small targets. Due to the low contrast between small-sized defects and the background, these defects are difficult to identify directly in images. Furthermore, small targets are prone to being filtered out in the connection layers of convolutional neural networks (CNN), leading to missed detections. To address this issue, this paper adopts a more advanced algorithm aimed at enhancing the model’s robustness to complex backgrounds and improving its ability to recognize small targets. This study proposes a steel surface defect detection algorithm based on YOLOv9, with the main contributions as follows:

1. The depthwise separable convolution (DSConv) module is introduced into the backbone network structure, decreasing the number of model parameters, and accelerating the model’s inference speed.
2. The C3 module is incorporated to combine low-level detailed information with high-level semantic information, enhancing the model’s adaptability to target scale variations and improving detection accuracy.
3. The bidirectional feature pyramid network (BiFPN) is integrated into the neck network structure, enabling the model to identify fine features of small targets, thereby improving its recognition capability.
4. The DySample upsampling operator is introduced, which optimizes the upsampling process of feature maps through a dynamic sampling mechanism, improving spatial resolution and better capturing detailed information.

## Related work

### Feature fusion methods in deep learning: evolution from FPN to BiFPN

In deep learning tasks such as object detection and image segmentation, feature fusion not only enhances the model’s ability to detect objects at different scales but also reduces redundancy among features, improving the model’s robustness and overall performance. Advancements in feature fusion techniques are among the core factors for improving model performance. Object detection requires models to process multi-scale feature information to accurately recognize and localize objects of various sizes. Low-level features, characterized by high-resolution spatial information, provide rich spatial details and positional information, making them effective for localizing small objects. However, these low-level features often lack deep semantic understanding and are prone to interference from background noise. In contrast, high-level features focus on more abstract semantic information, excelling at distinguishing object categories and handling complex backgrounds. Although their low resolution weakens their ability to capture fine details, the compression of image information allows them to more effectively represent critical content within the image. Therefore, effectively fusing these two types of features in deep learning models to leverage their strengths and compensate for their weaknesses has become a key technique for enhancing detection accuracy and robustness.

To address this challenge, researchers have proposed various feature fusion methods. Feature Pyramid Networks (FPN^24^) were among the earliest attempts to solve this problem. By adopting a top-down feature fusion structure, FPN combines high-level semantic information with low-level detailed information, significantly enhancing the model’s ability to detect objects at multiple scales. By gradually integrating deep high-level features into shallow high-resolution features, FPN achieves strong performance in detecting both large and small objects. Building on FPN, further improvements such as Path Aggregation Network (PANet^25^) introduced bottom-up path fusion and a global information propagation mechanism, enhancing the representational capacity of features. This design allows low-level features to more fully incorporate high-level semantic information, significantly improving detection performance in complex backgrounds and multi-scale object scenarios. The Bi-directional Feature Pyramid Network (BiFPN^26^) optimized the feature fusion process by introducing bi-directional flows and skip connections. BiFPN not only retains the multi-scale feature fusion advantages of FPN and PANet but also incorporates learnable feature weighting mechanisms to further improve fusion efficiency and detection accuracy. Compared to traditional methods, BiFPN achieves superior multi-scale detection capability in complex scenarios while maintaining high efficiency. These continuous innovations and improvements in feature fusion methods have led to significant advancements in the performance of object detection models across various application scenarios. However, with the growing demands of real-world applications, striking the optimal balance between efficiency and accuracy remains a critical direction for future research. Further optimizations may involve developing more efficient feature fusion strategies and smarter model designs to meet the needs of industrial applications.

### Upsampling techniques in deep learning

In the field of deep learning, particularly in tasks such as object detection and image segmentation, upsampling modules play a critical role. The core function of these modules is to enhance the resolution of feature maps, thereby reconstructing the detailed information of images, which is essential for accurately detecting and recognizing objects of varying scales. Upsampling techniques enlarge low-resolution feature maps to high resolution, enabling the model to capture image features more precisely, which is especially important for small object detection. During the upsampling process, it is crucial not only to upscale the feature maps but also to restore as much detailed information as possible, which significantly impacts the accuracy of tasks such as image segmentation. Different upsampling methods provide various trade-offs between computational efficiency and detail reconstruction capability. Selecting an appropriate upsampling technique is therefore critical for optimizing model performance and resource utilization.

Nearest Neighbor Interpolation is one of the simplest and most computationally inexpensive upsampling methods. It enlarges images by replicating the values of the nearest pixels, making it highly efficient for real-time detection tasks with strict processing speed requirements. While this method is easy to implement and fast, it performs poorly in reconstructing image details, often producing blocky artifacts. Its limitations are particularly evident in high-resolution or detail-rich scenarios, making it less ideal for tasks requiring fine-grained detail preservation. As a result, it is commonly used in applications where real-time performance is more critical than detail reconstruction. To address the limitations of simple upsampling methods, CARAFE (Content-Aware ReAssembly of FEatures)^27^ offers a more advanced upsampling strategy. Unlike fixed interpolation methods, CARAFE employs convolution operations to dynamically generate upsampling weights, adjusting the reassembly strategy based on the content of the input feature maps. CARAFE excels in adapting to complex backgrounds and multi-scale objects, reconstructing finer image details while effectively reducing artifacts and information loss during the interpolation process. Through its content-aware feature reassembly mechanism, CARAFE significantly improves model performance in many complex detection tasks. Dysample^28^, on the other hand, represents a novel upsampling approach that emphasizes dynamic learning and adaptability. Dysample dynamically adjusts upsampling parameters based on the characteristics of the input feature maps, making it particularly suitable for detecting small objects and handling complex scenes. Compared to static upsampling methods, Dysample is more flexible, enhancing detail reconstruction and detection accuracy without increasing computational overhead. However, its performance in certain specific scenarios, such as high-speed moving objects or dense object detection, may lack stability. Further research and optimization are needed to improve its consistency and applicability. These upsampling methods, through their respective innovations, have greatly enhanced the performance of deep learning models in object detection and image segmentation tasks. Future research directions may focus on developing more efficient upsampling techniques that are better suited to diverse scenarios, aiming to further improve detection accuracy and computational efficiency.

### YOLOv9 model

**Fig. 1:**
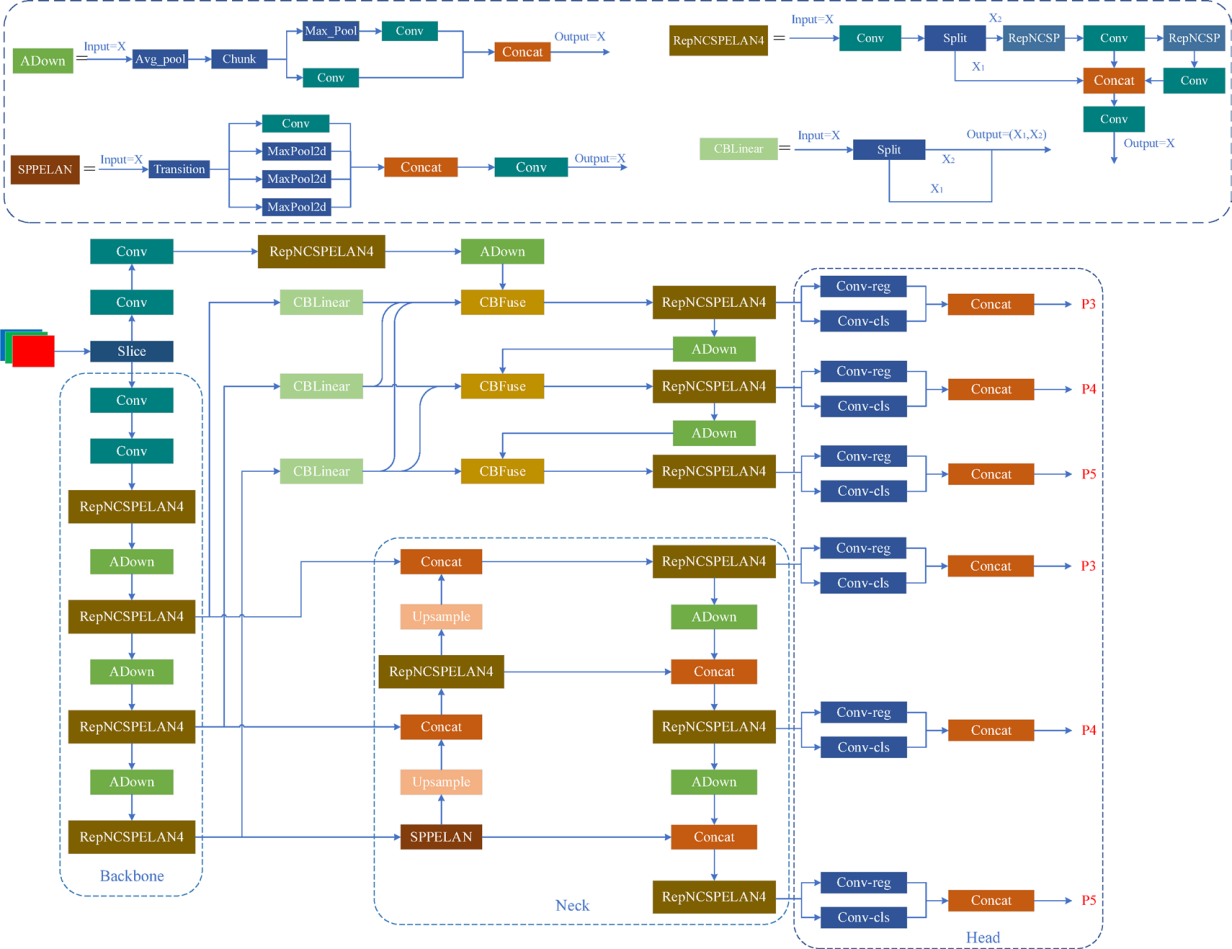
YOLOv9 network architecture diagram.

The YOLOv9 network structure, as shown in Fig. 1, consists of three main components: the Backbone, the Neck, and the Head. The Backbone is primarily composed of Conv and RepNCSPELAN4 modules. The RepNCSPELAN4 module cleverly combines the CSPNet structure from YOLOv4 with the ELAN structure from YOLOv7, effectively aggregating information within the network, reducing information loss during propagation, and significantly enhancing inter-layer information interaction. The Neck structure continues to adopt the FPN-PAN architecture for feature fusion, which efficiently integrates multi-scale feature information. The Head is responsible for equivalently fusing the convolutional features generated by the RepNCSPELAN4 module and accelerating the inference speed of the model. Additionally, three detection heads of different sizes are designed to identify large, medium, and small targets, enabling greater adaptability to variations in object size. This design ensures high accuracy across various complex environments.

## Improvements in YOLOv9 design concepts and network architecture

**Fig. 2:**
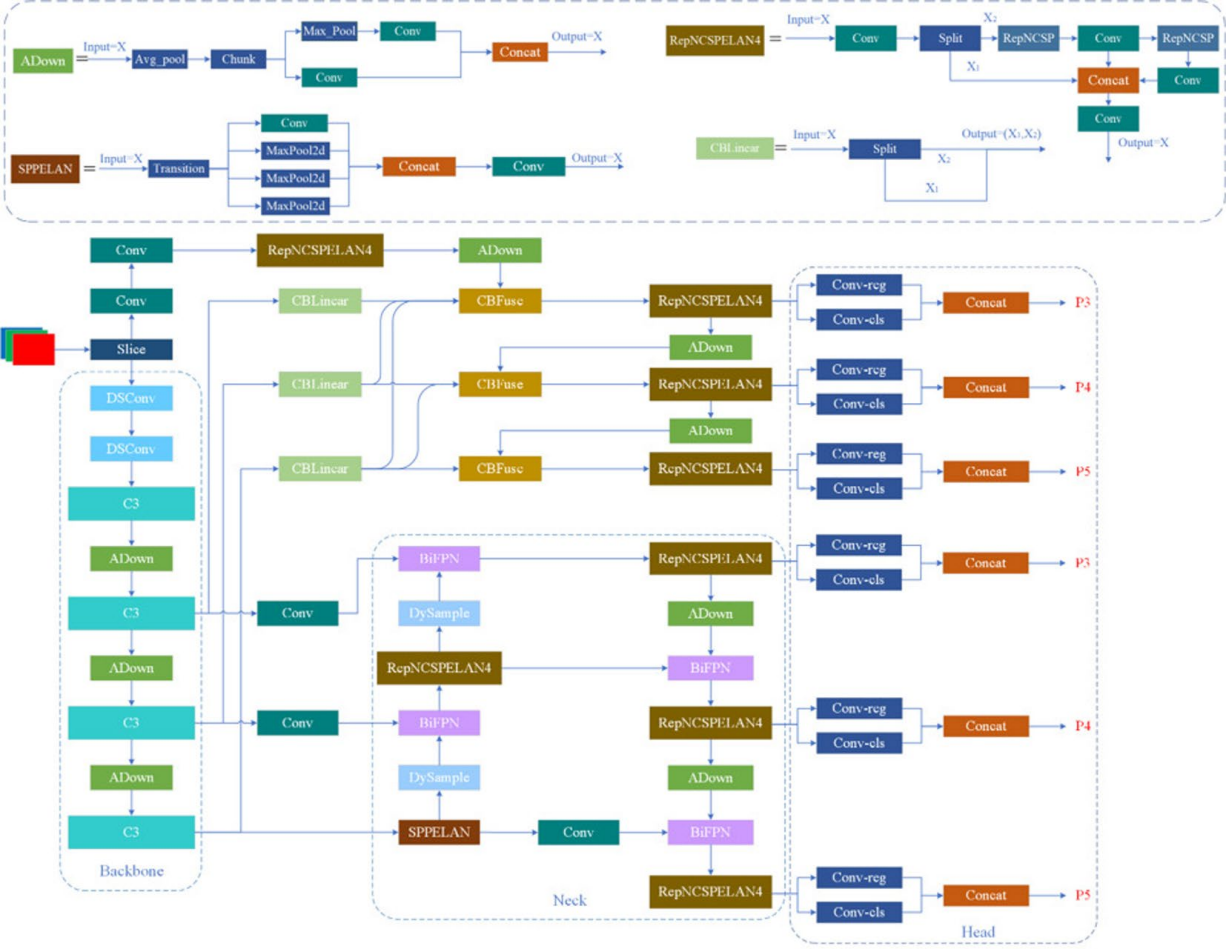
Improved YOLOv9 network architecture diagram.

In this paper, improvements were made to YOLOv9, as shown in Fig. 2. The introduction of deep separable Convolution (DSConv) into the backbone structure reduces the number of parameters, reduces the complexity of the model, and maintains the model detection performance. The C3 module was integrated into the backbone network, combining features from different network layers, enabling the model to better handle scale variations and thus enhance detection performance. In the neck network structure, the bidirectional feature pyramid network (BiFPN) was introduced, allowing the model to more accurately detect small objects in the image while reducing false positives and missed detections, further improving detection precision. Additionally, the DySample module was incorporated into the neck network structure to reduce sampling in background regions, enabling the model to more precisely locate and identify target regions, thereby enhancing the output performance of the model.

### Depthwise separable convolution

**Fig. 3:**
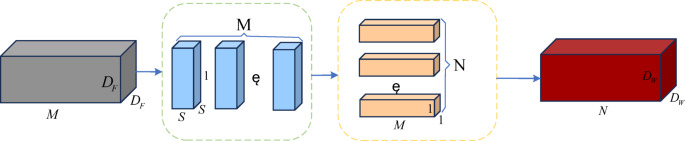
DSConv structure diagram.

During the model training process, an excessive number of convolutional layers often leads to a large number of parameters, which in turn consumes substantial computational resources and increases the model’s complexity^29^. This not only reduces the model’s detection capability but also negatively impacts its performance. This study employs Depthwise Separable Convolution (DSConv^30^), the structure of which is shown in Fig. 3.

The process of standard convolution: using* N * convolution kernels of size $S \times S$ and depth *M* to convolve the input feature map of size ${D_F} \times {D_F}$, resulting in * N * feature maps of size ${D_W} \times {D_W}$. The number of parameters is ${P_1}={S^2} \times M \times N \times D_{F}^{2}$.

The process of depthwise separable convolution: first,* M* convolution kernels of size $S \times S$ and depth 1 are used to perform depthwise convolution on the input feature map of size ${D_F} \times {D_F}$. Then,* N* convolution kernels of size 1 × 1 and depth *M* are applied to the result from the previous step through pointwise convolution, generating *N* feature maps of size ${D_W} \times {D_W}$. The number of parameters for DSConv is ${P_2}={S^2} \times M \times D_{F}^{2}+{\text{ }}M \times N \times D_{F}^{2}$, and the ratio of DSConv to standard convolution in terms of the number of parameters is:

1 $$\frac{{{P_2}}}{{{P_1}}}=\frac{{{S^2} \times M \times D_{F}^{2}+{\text{ }}M \times N \times D_{F}^{2}}}{{{S^2} \times M \times N \times D_{F}^{2}}}=\frac{1}{N}+\frac{1}{{{S^2}}}$$

In the formula, the ratio of ${P_2}$ to ${P_1}$ is much less than 1, indicating that using DSConv significantly reduces the model’s complexity and computational cost, while maintaining the network’s ability to capture spatial features and improving the model’s detection speed.

### C3 module

YOLOv9 still faces challenges in handling the diversity of defect scales. To address this issue, the C3 module was introduced into the backbone network to enhance the model’s ability to recognize defects of different scales. By leveraging an effective feature fusion strategy, the model can more accurately detect and locate targets, maintaining high detection accuracy even in complex scenarios.

The C3 module^31^, as an innovative component in deep learning architectures, shares a design philosophy similar to the classic CSP (Cross Stage Partial Network) architecture. Compared to the BottleneckCSP module, one notable difference in the C3 module lies in the choice of activation function: the C3 module employs the SiLU (Sigmoid-like Unit) function instead of the traditional LeakyReLU.

**Fig. 4:**
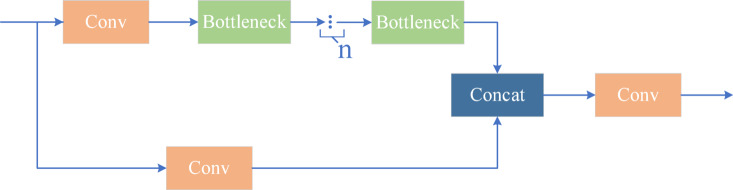
C3 structure diagram.

The structure of the C3 module, as shown in Fig. 4, consists of three convolutional modules and n BottleNeck modules. The details are as follows: the convolutional module in the main branch performs a 1 × 1 convolution operation with a stride of 1. The BottleNeck module features a dual-branch design. In the first branch, a 1 × 1 convolution first halves the number of channels in the feature map, followed by a 3 × 3 convolution that extracts features and doubles the number of channels again. The second branch connects via a shortcut, allowing for residual connections where the original input is added to the feature map processed by the convolution, achieving feature fusion. The auxiliary branch performs a 1 × 1 convolution operation, and the output is concatenated with the result from the main branch, after which another 1 × 1 convolution operation further integrates the feature information.

### Weighted bidirectional feature pyramid

**Fig. 5:**
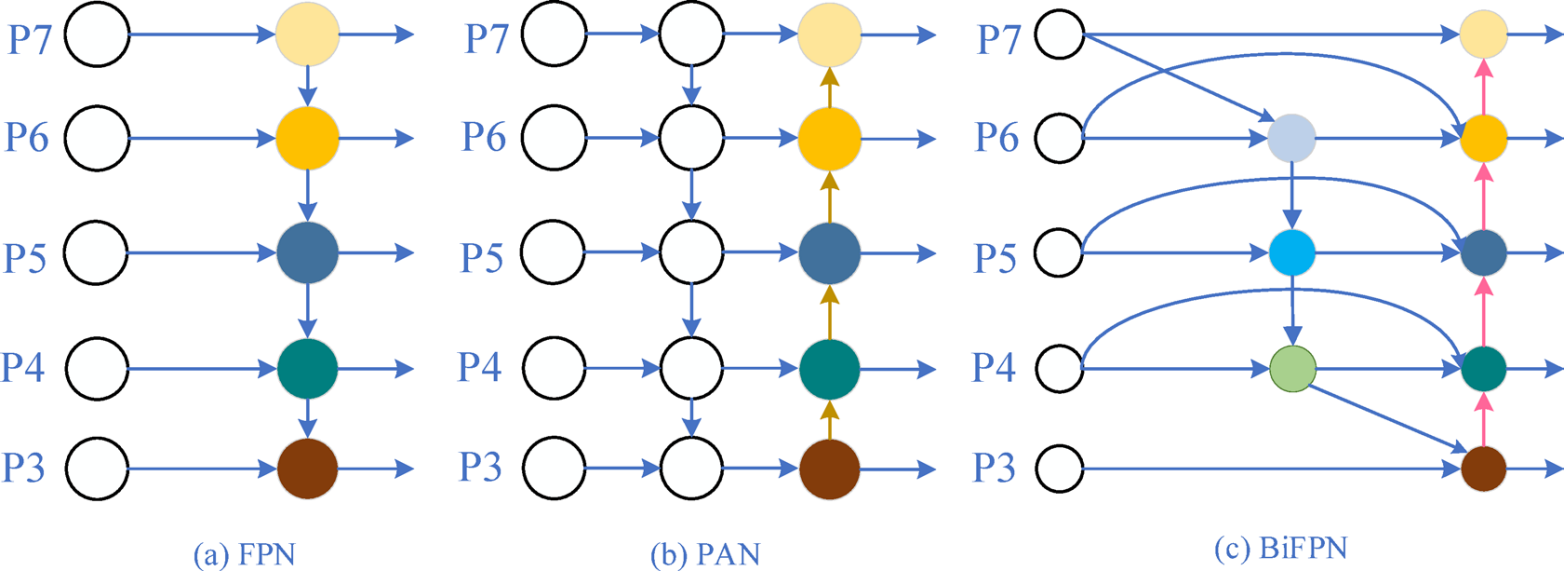
FPN, PAN, and BiFPN structure diagram.

As shown in Fig. 5a, the unidirectional structure of the Feature Pyramid Network (FPN) performs feature fusion through top-down channels. In Fig. 5b, the bidirectional structure of the Path Aggregation Network (PAN) adds bottom-up channels on top of FPN, enabling the transfer of low-level position information to higher layers, achieving bidirectional fusion of semantic and positional information. In traditional PAN, due to the inability to fully capture and utilize the original image features extracted by the backbone network, fine-grained low-level feature information is lost during the feature transfer process. This loss is especially significant for small objects with subtle defects. Such information loss not only affects the model’s recognition accuracy but also weakens the model’s ability to effectively detect small targets. To address this issue, this study introduces the Bidirectional Feature Pyramid Network (BiFPN), as shown in Fig. 5c, as an improvement to the YOLOv9 architecture. Compared to FPN and PAN, the main advantages of BiFPN are as follows: first, it removes input nodes with minimal contribution to feature fusion, such as the P3 and P7 nodes, thereby improving the feature fusion efficiency and reducing the network’s parameters and computational burden. Second, it introduces cross-scale connections to fuse features from different scales, enabling more precise target localization and recognition. Additionally, the algorithm incorporates a weighted feature fusion mechanism, assigning different weights based on the contribution of features at different levels, allowing the model to autonomously learn the weight values during training. To ensure the stability of the training process, a fast normalization fusion method based on SoftMax is proposed, ensuring the stability and optimal fusion of features from different scales during the feature fusion process. It is implemented using the following expression:

2 $$\it O=\sum\limits_{{\text{i}}} {\frac{{w_{i}}}{{\varepsilon +\sum\limits_{{\text{i}}} {w_{j}} }}} \cdot I_{i}$$

, where ${I_i}$ represents the feature at the *i*-th level, ${w_i}$ is the corresponding weight of the feature, and $\varepsilon =0.0001$ is used to avoid instability in the values. All weight values are constrained between 0 and 1, reflecting the varying importance of different inputs.

### DySample upsampling operator

In the YOLOv9 model, the upsampling operation originally used the nearest neighbor interpolation method, which achieves fast upsampling by directly setting the value of the new pixel to that of its nearest neighbor. Although this method has an advantage in speed, it often lacks sufficient noise resistance when handling complex background noise, affecting the model’s accuracy in identifying target areas. To address the insufficient noise resistance of the nearest neighbor interpolation method when dealing with complex background noise, the lightweight DySample dynamic upsampling module was introduced as an improvement. This module enhances the model’s ability to recognize target areas by optimizing the distribution of sampling points and effectively reduces interference from background noise. Additionally, the DySample module reduces the number of model parameters and computational complexity, improving the accuracy and robustness of target detection, making it more suitable for industrial real-time detection applications.

**Fig. 6:**
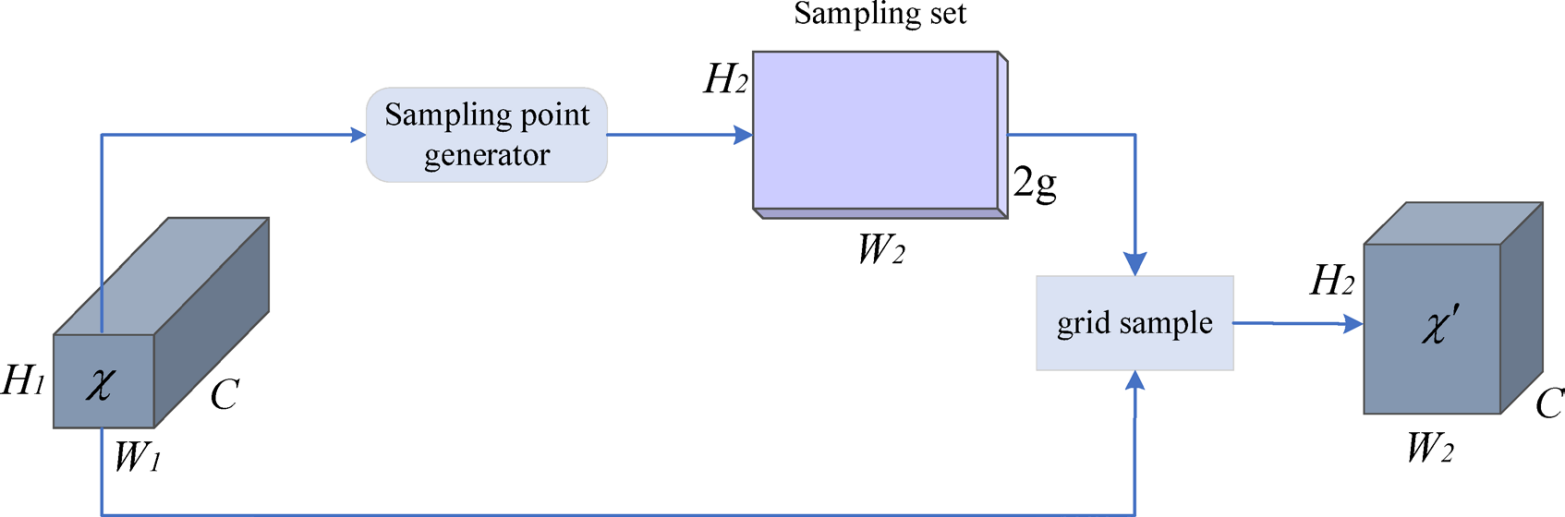
DySample module for dynamic upsampling.

The main process of upsampling using the DySample module is shown in Fig. 6. Given a feature map $\chi$ of size $C \times {H_1} \times {W_1}$ and a point sampling set $\Omega$ of size $2g \times {H_2} \times {W_2}$, where the first dimension $2g$ represents the x and y coordinates, the grid_sample function uses the positions in the point sampling set $\Omega$ to resample the feature map $\chi$, generating a result $\chi ^{\prime}$  of size $C \times {H_2} \times {W_2}$, as shown in Eq. (3):

3 $$\chi ^{\prime}=grid\_sample\left( {\left. {\chi ,\Omega } \right)} \right.$$

**Fig. 7:**
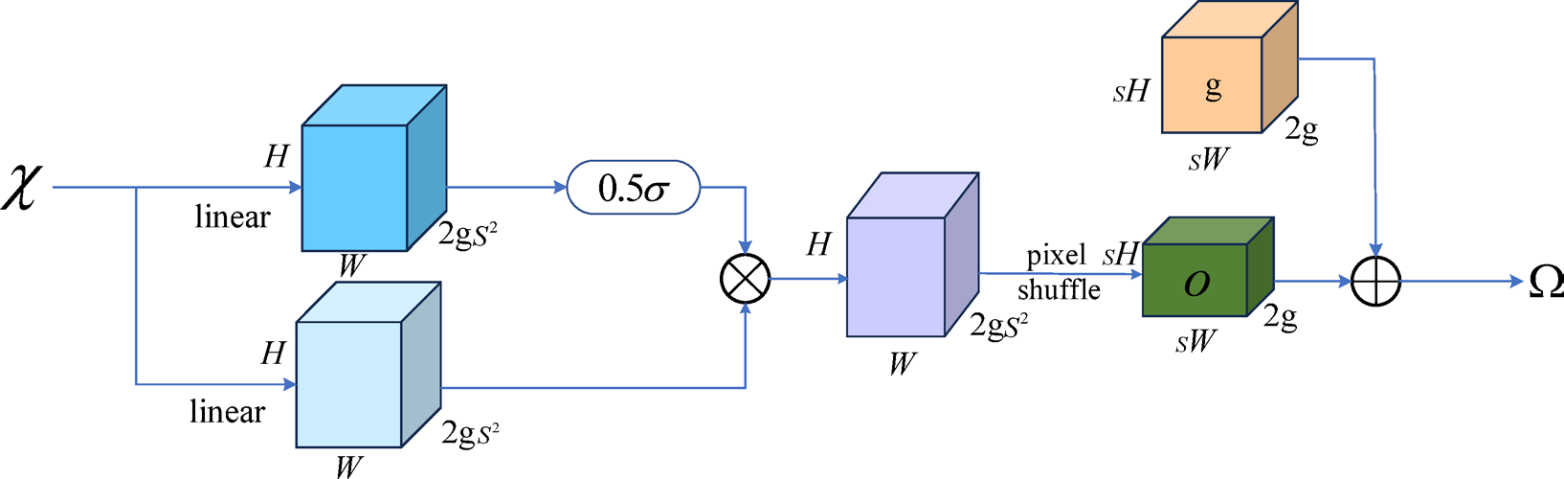
Point sampling based on dynamic range factor.

As shown in Fig. 7, first, given an upsampling scale factor *S *and a feature map $\chi$ of size $C \times H \times W$, a linear layer is used to generate an offset* O* of size $2g{s^2} \times H \times W$. The input channel number of this linear layer is* C*, and the output channel number is $2g{s^2}$. Next, through Pixel Shuffle, the offset * O *is reshaped to size $2g \times sH \times sW$. Finally, the point sampling set $\Omega$ is the sum of the offset * O *and the original sampling grid* g*, as shown in Eqs. (4) and (5):

4 $$O=linear\left( \chi \right)$$

5 $$\Omega =O+g$$

## Experiments

### Dataset

**Fig. 8:**
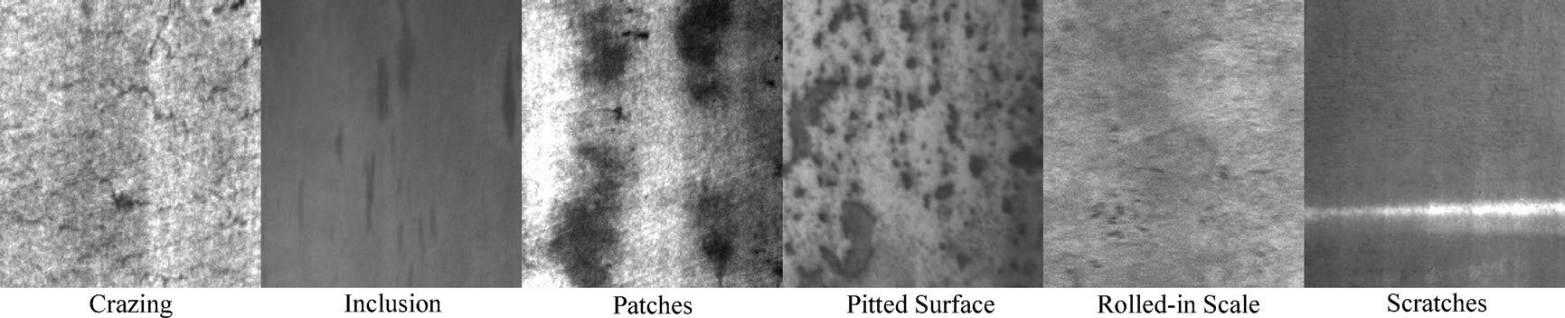
Six types of defect samples.

This paper utilizes the publicly available NEU-DET steel surface defect dataset from Northeastern University for training and validation. The dataset contains six main types of defects found on steel surfaces: inclusion, scratches, crazing, patches, rolled-in scale, and pitted surface. Each defect type has 300 images, for a total of 1800 images. The images are 200 × 200 pixels in size, with each image showcasing different types of defects on steel surfaces. The image backgrounds are typically uniform steel surfaces, while the defects are displayed with varying contrasts, textures, and shapes. For rolled-in scale defects, the images show scaly, irregular dark patches or streaks on a uniform background. For patches defects, the images depict small areas of scratches or worn-out regions on the steel surface, which are slightly darker than the background and have irregular shapes resembling small spots. Crazing defects are represented as elongated lines or mesh-like structures, which appear darker and more abrupt than the surrounding areas, showing clear lines or cracks. Inclusion defects appear as extrusion defects formed during the manufacturing process, typically showing as indented or raised areas contrasting with the smooth background, with irregular edges and sometimes variations in grayscale levels. Scratches defects are shown as elongated lines parallel to the surface, slightly darker than the background, arranged uniformly or with some directionality. Pitted surface defects display indentations or small pits on the steel surface, appearing as dark dots or irregular shapes contrasting with the smooth background. Based on the dataset split ratio, the dataset is divided into training, testing, and validation sets in a 7:2:1 ratio, resulting in 1260 training images, 360 testing images, and 180 validation images. The six types of defect samples are shown in Fig. 8.

### Experimental environment and parameter settings

In this experiment, the training was conducted using a GPU (RTX 4090 (24GB)), a CPU (16vCPU Intel^®^ Xeon^®^ Platinum 8352 V CPU @ 2.10 GHz), and CUDA 11.3. The experimental environment’s operating system was Ubuntu 20.04, with the PyTorch 1.11.0 deep learning framework, and Python 3.8 was used for programming. During the training process, the pre-trained model “yolov9-c.pt” was loaded. The resolution of the training images was set to 640 × 640, the batch size was set to 4, the number of epochs was 300, workers was set to 8, the learning rate was 0.01, the momentum parameter was 0.937, and the weight decay coefficient was 0.0005.

### Evaluation metrics

In this study, the evaluation metrics include the F1 score, Precision (P), Recall (R), Average Precision (AP), and mean Average Precision (mAP)^32^. Additionally, the number of parameters, frames per second (FPS), and giga floating-point operations per second (GFLOPS) are also considered as reference metrics. The corresponding formulas for the evaluation metrics are presented as follows:

6 $$P=\frac{{T_{p}}}{{T_{p}+F_{p}}}$$

7 $$R=\frac{{T_{p}}}{{T_{p}+F_{N}}}$$

8 $$AP=\int_{0}^{1} {P(R) {\text{d}}R}$$

9 $$\it {\text{m}}AP=\frac{1}{n}\sum\limits_{{{\text{i}}=0}}^{n} {AP(i)}$$

10 $${\text{F}}1=\frac{{2 \times {\text{Precision}} \times {\text{Recall}}}}{{{\text{Precision}}+{\text{Recall}}}}$$

, where $T_{p}$ represents the number of correctly detected defective objects, $F_{p}$ represents the number of incorrectly detected defective objects, $F_{N}$ represents the number of missed defective objects, *n* represents the number of defect categories, and $AP(i)$ denotes the average precision for the *i*-th object class.

## Model comparison and evaluation

### Ablation study on convolution methods

In the field of machine learning and deep learning, convolution operations are widely adopted due to their unique advantages in capturing local patterns and features within input data. Convolution not only efficiently extracts local features from images but also has translation invariance, enabling the model to recognize the same pattern in different positions of the image, which is crucial for improving model accuracy. To further enhance object detection performance, this paper introduces three types of convolutions: Spectral-Channel Convolution (SCConv^33^), lightweight Ghost Convolution (GhostConv^34^), and Depthwise Separable Convolution (DSConv). To evaluate the performance and select the best convolution, the Conv modules in the Backbone of YOLOv9 were replaced, and experiments were conducted on the publicly available NEU-DET dataset to compare performance. The results are shown in Table 1.

**Table 1:** Ablation study results on different convolution methods.

Number	Module Name	Precision/%	Recall/%	mAP@0.5/%	FPS(f/s)	GFLOPS	Params(M)
1	Conv	75.1	69.8	76.4	71.4	236.7	50.70
2	SCConv	74.0	70.6	76.1	59.5	244.8	50.78
3	GhostConv	79.8	66.5	76.6	72.2	234.7	50.67
4	DSConv	79.4	71.5	77.9	73.4	233.0	50.63

Table 1 shows that among the three convolution methods compared, their performance differs significantly. DSConv achieves the best performance in terms of mAP@0.5, reaching 77.9%, which is a 1.5% improvement compared to the baseline model using the Conv module (76.4%). Additionally, it achieves a significant improvement in Precision, reaching 79.4%, which is 4.3% higher than the baseline model. Meanwhile, the parameter count is reduced to 50.63 M, representing a reduction of 0.14%, thus achieving model lightweighting. Moreover, DSConv demonstrates an inference speed of 73.4 FPS, outperforming GhostConv’s 72.2 FPS, showcasing superior computational efficiency. In contrast, GhostConv further reduces the parameter count to 50.67 M, but its Recall is only 66.5%, which is 3.3% lower than the baseline model and 5.0% lower than DSConv. SCConv shows some improvement in Recall, reaching 70.6%, but its mAP@0.5 is slightly lower at 76.1%, and its inference speed significantly drops to 59.5 FPS. Overall, DSConv achieves the best balance between precision, recall, and parameter count, significantly outperforming the other convolution modules in terms of mAP@0.5 and Precision while maintaining a low parameter count. This demonstrates its outstanding overall performance. Consequently, this study ultimately selects the DSConv module, which effectively reduces the number of model parameters and computational burden, significantly improving the model’s accuracy and efficiency, thereby providing an optimal solution for lightweight object detection tasks.

### Ablation study on module structure

In the YOLOv9 object detection framework, the original RepNCSPELAN4 module has certain limitations in feature extraction. To improve the quality of feature extraction and the performance of object detection, this paper proposes the introduction of the C3 module to obtain higher-quality feature maps. To validate the effectiveness of the proposed improvement, we introduced the following three feature extraction modules for comparison experiments: HGBlock^35^, C2f^36^, and C3. The results are shown in Table 2.

**Table 2:** Ablation study results on module structure.

Number	Module Name	Precision/%	Recall/%	mAP@0.5/%	FPS(f/s)	GFLOPS	Params(M)
1	RepNCSPELAN4	75.1	69.8	76.4	71.4	236.7	50.70
2	HGBlock	78.5	71.0	76.6	77.2	224.4	48.24
3	C2f	75.5	70.1	77.3	59.1	395.5	66.21
4	C3	71.6	76.6	78.5	75.1	230.1	49.82

The experimental results indicate that, compared to the baseline module RepNCSPELAN4, the C3 module improves the Recall by 6.8% and the mAP@0.5 by 2.1%. Although there is a slight decrease in Precision, the significant improvements in Recall and mean Average Precision effectively reduce missed detections, making it suitable for multi-object detection and complex scene applications. Meanwhile, compared to the C2f and HGBlock modules, the C3 module achieves a good balance between performance and computational efficiency, enhancing feature extraction capability and improving the detection performance of the model. With a relatively low GFLOPS (230.1) and parameter count (49.82 M), the inference speed of the C3 module reaches 75.1 FPS, demonstrating excellent real-time performance. Comprehensive analysis shows that the C3 module offers significant advantages in feature extraction quality and object detection performance. Consequently, it was ultimately selected as the feature extraction module for the YOLOv9 framework to achieve higher-quality object detection outcomes.

### Ablation study on network structure

To evaluate the impact of the improved module on the detection performance of the publicly available NEU-DET dataset, this study employed a network structure ablation experiment to visually assess the effect of each module. By comparing the detection performance of network structures with different modules on the NEU-DET dataset, we can systematically analyze the contribution of each module to the overall performance. The experimental results are summarized in Table 3, which provides performance metrics under various network configurations to help understand the practical effectiveness of the improved module.

**Table 3:** Ablation results of network structure.

Number	Experiments	Precision/%	Recall/%	mAP@0.5/%	FPS (f/s)	GFLOPS	Params (M)
1	YOLOv9	75.1	69.8	76.4	71.4	236.7	50.70
2	YOLOv9 + DSConv	79.4	71.5	77.9	73.4	233.0	50.63
3	YOLOv9 + DSConv + C3	78.1	71.3	78.1	75.1	229.8	49.75
4	YOLOv9 + DSConv + C3 + BiFPN	82.3	67.1	77.7	80.2	210.3	46.15
5	YOLOv9 + DSConv + C3 + BiFPN + Dysample	82.5	68.4	78.2	79.3	212.3	46.16

Table 3 presents the ablation study results of the YOLOv9 network structure, where the performance and computational efficiency of the model are evaluated by progressively introducing the DSConv, C3, BiFPN, and Dysample modules. The baseline model, YOLOv9, achieves an mAP@0.5 of 76.4%, an inference speed of 71.4 FPS, and a parameter count of 50.70 M, demonstrating good overall performance. After incorporating the DSConv module, the model’s Precision significantly increases to 79.4%, while Recall and mAP@0.5 improve to 71.5% and 77.9%, respectively. Additionally, inference speed (FPS) and computational complexity are optimized. With the further addition of the C3 module, mAP@0.5 increases to 78.1%, FPS improves to 75.1 frames per second, and both GFLOPS and parameter count are reduced, indicating that the C3 module enhances feature extraction capabilities significantly through its cross-stage feature connection mechanism. On this foundation, the introduction of the BiFPN module improves Precision to 82.3% and inference speed to 80.2 FPS, though Recall decreases slightly, suggesting that while BiFPN enhances detection precision, it may impact the recall ability. Finally, with the addition of the Dysample module, Precision further improves to 82.5%, Recall recovers to 68.4%, mAP@0.5 increases to 78.2%, and inference speed remains at a high level of 79.3 FPS. Meanwhile, the parameter count and computational complexity are maintained within reasonable ranges (46.16 M and 212.3 GFLOPS). The comprehensive results demonstrate that by progressively optimizing the combination of modules, YOLOv9 achieves a well-balanced trade-off between performance, efficiency, and complexity in object detection tasks, showcasing its powerful detection capabilities.

### Performance comparison of different models

To demonstrate the effectiveness of the performance improvements in the proposed model, our model was compared with nine mainstream object detection models, including SSD, Faster R-CNN, and YOLO series models, under the same dataset and training iterations. The results are shown in Table 4. According to the algorithm comparison results in Table 4, the algorithms exhibit significant differences in Precision, mAP@0.5, FPS, GFLOPS, and the number of parameters. These differences provide valuable insights for an in-depth analysis and comparison of the applicability of each algorithm.

**Table 4:** Algorithm comparison experiment results.

Algorithm	Precision/%	mAP@0.5/%	FPS(f/s)	GFLOPS	Params(M)
SSD	70.2	68.6	–	–	–
Faster R-CNN	79.9	77.5	–	–	–
YOLOv5s	71.7	75.2	208.3	15.8	7.02
YOLOv7	63.3	71.7	109.8	103.2	36.50
YOLOv8s	75.7	71.7	312.5	28.4	11.12
YOLOv8n	69.4	73.3	232.5	8.1	3.00
YOLOv9	75.1	76.4	71.4	236.7	50.70
YOLOv10n	73.3	74.9	294.1	8.2	2.69
YOLOv11n	73.3	77.8	416.6	6.3	2.58
Ours	82.5	78.2	79.3	12.3	46.16

From the experimental results, our proposed model achieved superior performance across multiple key metrics. Specifically, the Precision reached 82.5%, and the mAP@0.5 achieved 78.2%, the highest values among all the compared models. These results significantly surpass the baseline model YOLOv9, which recorded a Precision of only 75.1% and an mAP@0.5 of 76.4%. This indicates that the improved feature extraction module and network structure played a crucial role in enhancing detection accuracy. Additionally, the inference speed of the proposed model reached 79.3 FPS, achieving good real-time performance while maintaining high accuracy. Although the inference speed is lower than YOLOv11n’s 416.6 FPS, it still demonstrates excellent overall performance. Compared to other models, classic object detection algorithms such as SSD and Faster R-CNN showed relatively weaker performance. For example, SSD achieved an mAP@0.5 of only 68.6%, the lowest among all compared models, and also exhibited poor real-time performance, making it unsuitable for detection tasks in complex scenarios. Although Faster R-CNN achieved an mAP@0.5 of 77.5%, close to that of the YOLO series models, its low inference speed makes it inadequate for tasks requiring high real-time performance. Within the YOLO series models, YOLOv8s and YOLOv11n demonstrated high inference speeds of 312.5 and 416.6 FPS, respectively. However, their accuracy lags behind the proposed model (YOLOv8s: mAP@0.5 of 71.7%, YOLOv11n: mAP@0.5 of 77.8%). Other models such as YOLOv5s, YOLOv8n, and YOLOv10n showed certain advantages in terms of accuracy and real-time performance, but their overall performance was still inferior to the proposed improved model. In terms of GFLOPS and parameter count, the proposed model achieved optimizations in computational complexity and storage requirements while maintaining high accuracy. Compared to the baseline model YOLOv9, the GFLOPS decreased from 236.7 to 212.3, and the parameter count was reduced from 50.70 M to 46.16 M, demonstrating excellent lightweight characteristics. In summary, the improved YOLOv9 model not only maintains high detection accuracy but also further optimizes inference speed and lightweight design, showcasing superior overall performance.

## Result visualization

We presented the performance of various algorithms in detecting different types of surface defects, clearly illustrating the detection capabilities and accuracy of each algorithm through a series of visualized images. These images intuitively show the strengths and weaknesses of the algorithms in identifying various surface defects, such as (a) cracks, (b) inclusions, (c) patches, (d) pitting surfaces, (e) rolled scale, and (f) scratches. By comparing the detection results of different algorithms, we can more intuitively understand the effectiveness and performance differences of each algorithm in practical applications, providing an important basis for further optimization and selection of appropriate algorithms.

**Fig. 9:**
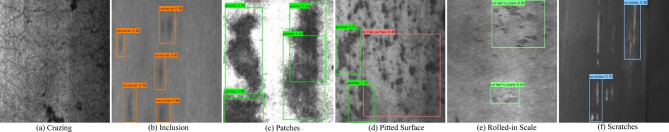
SSD algorithm.

Based on the visualized results of the SSD algorithm in Fig. 9, the algorithm shows varying performance in detecting different types of surface defects. In crack detection, the algorithm failed to effectively recognize the defect, performing poorly. In detecting inclusions, it accurately identified the defect category. For patches, the algorithm performed well, correctly marking the defects. However, in detecting pitted surfaces, the algorithm displayed two false positives for patch defects, showing some errors. When detecting rolled-in scale, the detection confidence was low. In scratch detection, the algorithm was fairly accurate, but it missed some fine scratches. Overall, the SSD algorithm works well for detecting prominent defects but struggles with more complex defects.

**Fig. 10:**
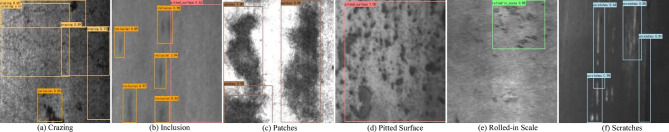
Faster R-CNN algorithm.

Based on the visualized results of the Faster R-CNN algorithm in Fig. 10, the algorithm’s performance in detecting different surface defects is as follows: in detecting cracks and inclusions, the algorithm identified multiple potential defect areas, but with some false positives; for patches, the detection was effective, accurately identifying most patch defect areas; in pitted surface detection, the algorithm recognized the main areas but was not precise enough in detecting finer pits; for rolled-in scale detection, the algorithm identified some regions, but overall performance was average, with some missed detections; in scratch detection, the algorithm performed well, accurately marking several scratches, though it missed some fine scratches. Overall, the Faster R-CNN algorithm excels in detecting prominent defects but struggles with finer defects.

**Fig. 11:**
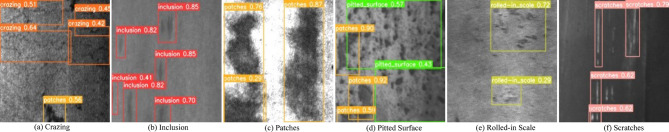
YOLOv5s algorithm.

Figure 11 shows the performance of the YOLOv5s algorithm in detecting different surface defects. In crack detection, the algorithm was able to identify multiple crack areas, but with some false positives; for inclusion detection, it marked several defect locations. In patch detection, the algorithm performed well, effectively identifying large patch areas, though the confidence for some results was low. In pitted surface detection, the algorithm identified the pitted areas but also falsely detected some patches. The performance in detecting rolled-in scale was average, with some inaccuracies. For scratch detection, the algorithm was fairly accurate, but the detection precision and consistency may be lacking. Overall, YOLOv5s performs well in detecting prominent and large-area defects, but struggles with finer defects.

**Fig. 12:**
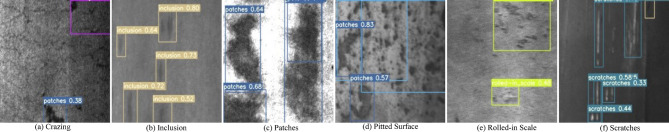
YOLOv7 algorithm.

Figure 12 shows the performance of the YOLOv7 algorithm in detecting various surface defects. In several detection tasks, YOLOv7 failed to accurately identify and localize defects, particularly when handling complex backgrounds and small defects, where it performed poorly. For example, in crack detection, YOLOv7’s localization was not precise, and some defect areas were missed. In detecting rolled-in scale, inclusions, patches, and scratches, YOLOv7 also showed insufficient detection accuracy and poor consistency. In contrast, YOLOv5s performed better in these tasks, accurately identifying and localizing defects. Therefore, although YOLOv7 may have certain advantages in other tasks, its performance in steel surface defect detection is significantly inferior to YOLOv5s.

**Fig. 13:**
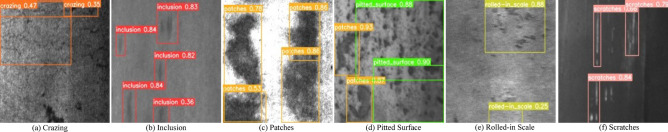
YOLOv8s algorithm.

Figure 13 shows the performance of the YOLOv8s algorithm in detecting different surface defects. For cracks and inclusions, the algorithm was able to identify some potential locations, but with low confidence and some missed detections. In patch detection, the algorithm performed well, effectively recognizing large patch areas, although some results had low confidence. In pitted surface detection, the algorithm accurately detected multiple regions with high confidence in the detection boxes, but there were still some false positives. The performance in detecting rolled-in scale was average, with low confidence in some areas. For scratch detection, the algorithm performed well, detecting scratches, though with low confidence in some cases and some missed detections. Overall, YOLOv8s performs well in detecting prominent defects but has some issues when handling complex crack-like defects.

**Fig. 14:**
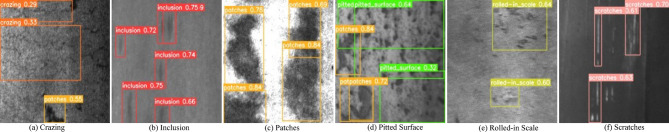
YOLOv8n algorithm.

Figure 14 shows the performance of the YOLOv8n algorithm in detecting various surface defects. Compared to the YOLOv8s algorithm, YOLOv8n performs slightly worse in terms of detection accuracy and confidence. YOLOv8n demonstrates lower confidence in detecting defects like cracks, inclusions, patches, and scratches, particularly when handling complex or fine defects, where it underperforms compared to YOLOv8s. YOLOv8s shows more accurate and reliable results, especially when detecting large-area defects such as patches and scratches. The strength of YOLOv8n lies in its lighter architecture, making it suitable for resource-constrained environments or scenarios that require faster speeds, though it falls short of YOLOv8s in terms of accuracy.

**Fig. 15:**
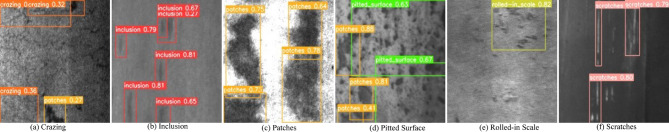
YOLOv9 algorithm.

Figure 15 shows the performance of the YOLOv9 algorithm in detecting various surface defects. In crack detection, the algorithm shows some false positives and missed detections, with low confidence levels. For inclusion detection, some of the detection boxes have higher confidence. In patch detection, the algorithm can identify large patch areas, though with lower confidence values. In pitted surface detection, the algorithm detects most regions, but some have low confidence, and there are false positives for patch-like defects. The performance in detecting rolled-in scale is average, with low confidence in some detections and some missed areas. In scratch detection, the algorithm is relatively accurate but still has a risk of missed detections. Overall, YOLOv9 performs well in detecting prominent defects.

**Fig. 16:**
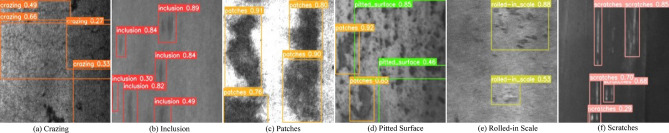
Our algorithm.

Figure 16 shows the performance of our algorithm in detecting various surface defects. Compared to the YOLOv9 algorithm, our algorithm performs significantly better in detecting all types of surface defects. For cracks and inclusions, our algorithm provides higher confidence in the detection boxes, with fewer false positives and missed detections. For patches and pitted surfaces, our algorithm more accurately identifies defect areas with higher confidence, demonstrating stronger detection capabilities. In detecting rolled-in scale, our algorithm is more precise and also has higher detection confidence. For scratches, our algorithm also performs exceptionally well, with better accuracy and confidence compared to YOLOv9. Overall, our algorithm exhibits higher accuracy and confidence across all defect types, with a lower false detection rate and stronger detection capabilities than the YOLOv9 algorithm.

**Fig. 17:**
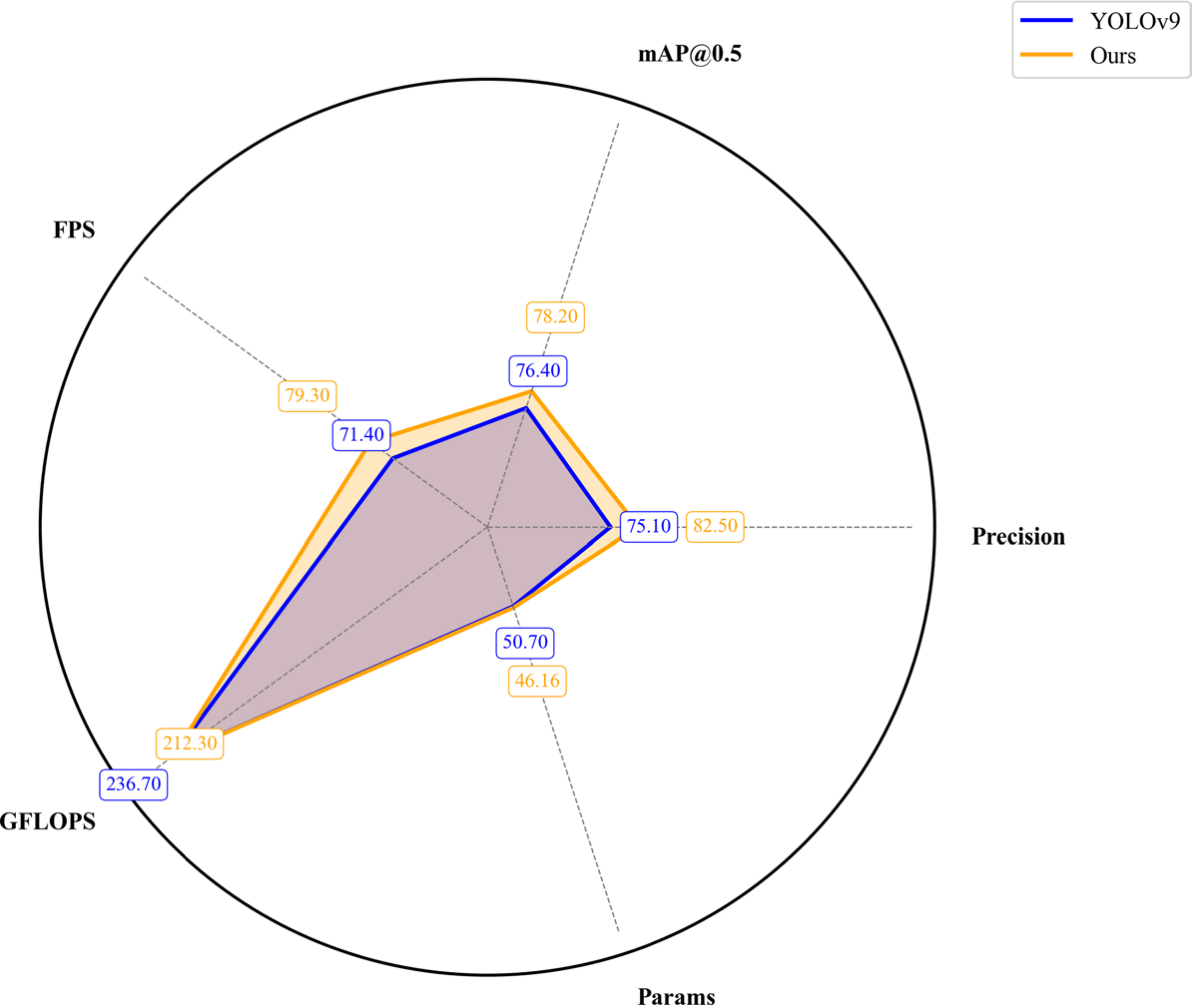
Performance comparison between our and YOLOv9 models.

To validate the performance advantages of our proposed model, a comparative experiment was conducted with the original model YOLOv9, and the results are shown in Fig. 17. Our model achieved an mAP@0.5 of 78.20%, which is 1.80% higher than YOLOv9, demonstrating superior detection accuracy. In terms of Precision, our model reached 82.50%, outperforming YOLOv9’s 75.10%. Regarding model complexity, the parameter count of our model is 46.16 M, significantly smaller than YOLOv9’s 50.7 M, representing an 8.90% reduction in parameter count and a substantial decrease in model complexity. The computational cost is 212.3 GFLOPS, approximately 10.30% lower than YOLOv9’s 236.7 GFLOPS. Meanwhile, our model achieved an inference speed of 79.30 FPS, approximately 9.96% faster than YOLOv9’s 71.40 FPS. In summary, our model achieves a well-balanced trade-off between detection performance, computational efficiency, and inference speed, surpassing the original model YOLOv9.

**Fig. 18:**
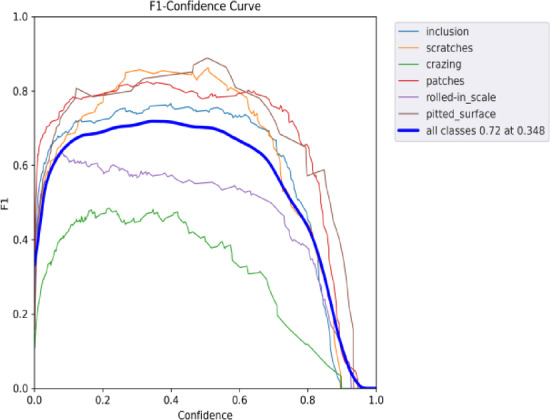
F1-confidence curve of YOLOv9.

**Fig. 19:**
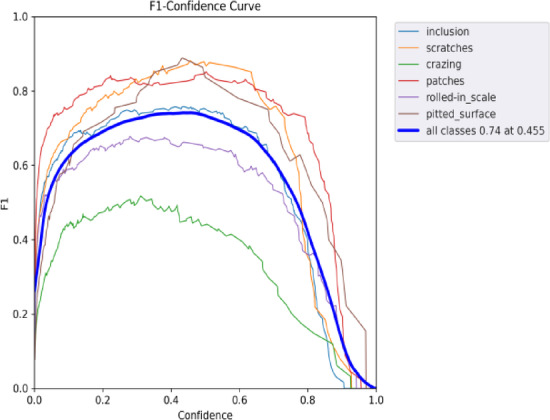
F1-confidence curve of the improved YOLOv9.

Figures 18 and 19 illustrate the F1-Confidence curves for the original algorithm and the improved algorithm across different target detection tasks. The improved YOLOv9 algorithm demonstrates significantly better performance on the F1-Confidence curve compared to the original YOLOv9 algorithm. The overall F1 score peak increased from 0.72 to 0.74, and the optimal confidence threshold rose from 0.348 to 0.455, indicating that the improved algorithm exhibits greater accuracy and robustness for high-confidence detection results. Among the individual categories, the scratches category showed the best performance, with the peak F1 score further improved after the modification, and the curve became smoother and more stable. For categories such as pitted_surface and patches, there were noticeable improvements in the medium to high confidence range. Overall, the improved YOLOv9 algorithm exhibited higher precision and stability across all categories, with particularly superior performance in the high-confidence range.

**Fig. 20:**
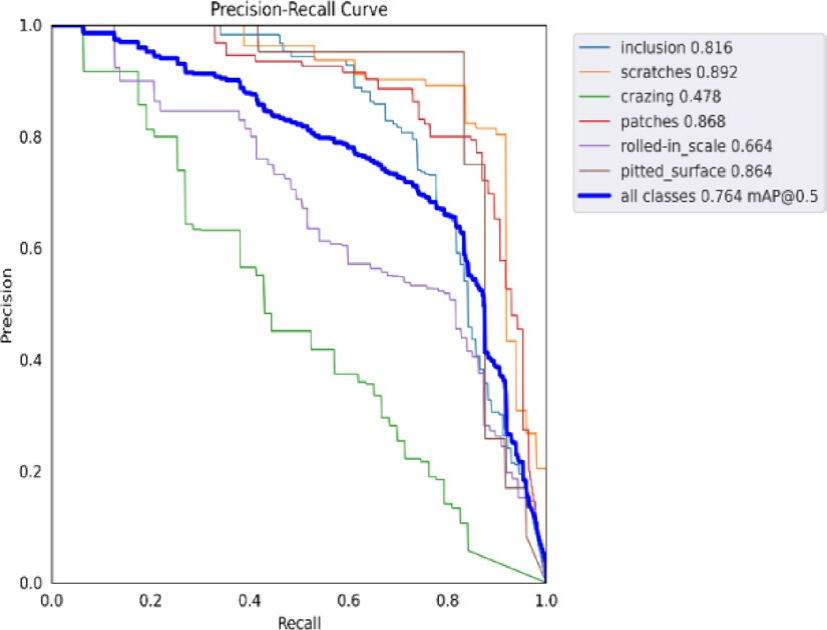
Precision-recall curve of YOLOv9.

**Figs. 21:**
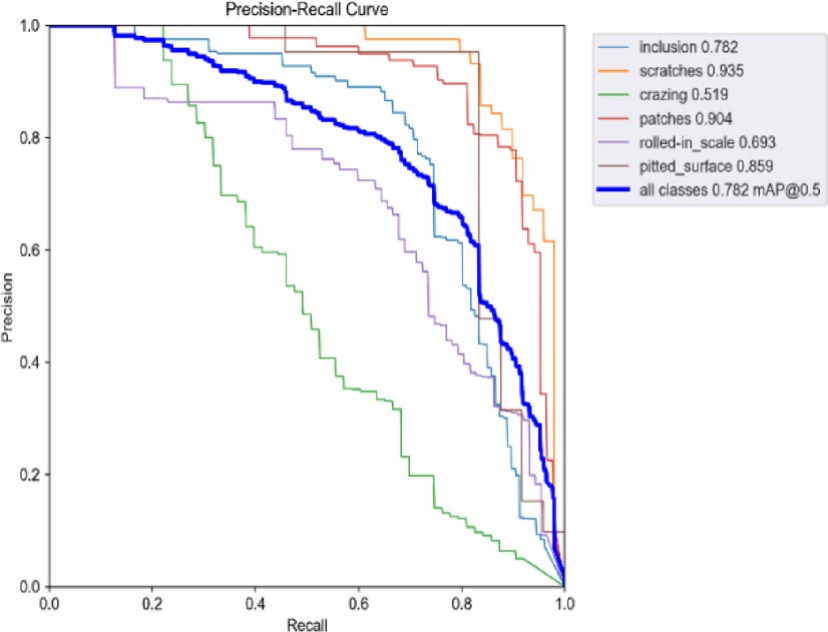
Precision-recall curve of the improved YOLOv9.

Figures 20 and 21 present the Precision-Recall curves of the original and improved algorithms for target detection tasks across different categories. When the IoU threshold is set to 0.5, the Precision-Recall curves and AP values for each category in the dataset are clearly displayed. It can be observed that the improved algorithm significantly outperforms the original algorithm in multiple categories. For instance, the AP values for the scratches and patches categories increased by 4.3% and 3.6%, respectively. Additionally, for the rolled-in scale and crazing categories, although the AP values of the improved algorithm only increased by 2.9% and 4.1%, respectively, the overall performance still surpasses the baseline algorithm. Overall, the improved algorithm enhances the model’s comprehensive performance through more accurate feature fusion and optimization strategies. The improved algorithm demonstrates higher model precision, thereby validating its effectiveness in practical applications.

**Fig. 22:**
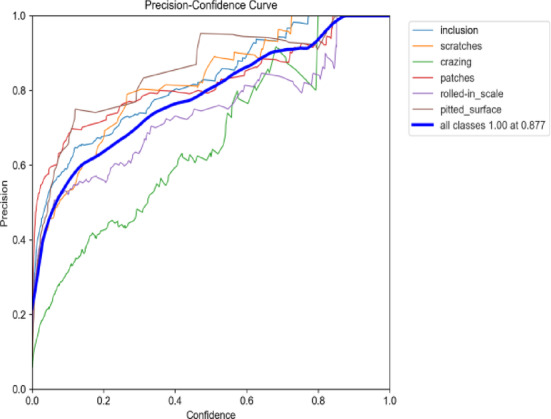
Precision-confidence curve of YOLOv9.

**Fig. 23:**
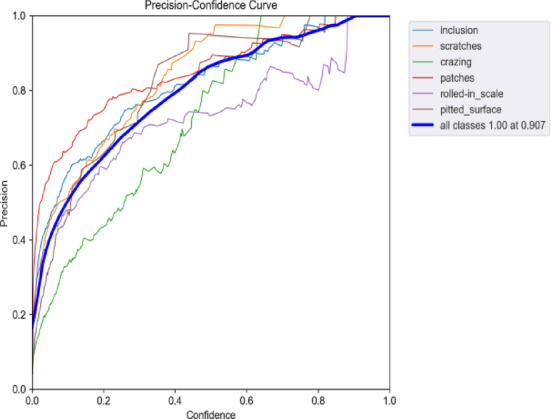
Precision-confidence curve of the improved YOLOv9.

Figures 22 and 23 illustrate the Precision-Confidence curves for the original algorithm and the improved algorithm across different target detection tasks. The improved YOLOv9 algorithm demonstrates significantly better performance on the Precision-Confidence curve compared to the original YOLOv9 algorithm. While the overall precision peak remains at 1.00, the optimal confidence threshold increased from 0.877 to 0.907, indicating that the improved algorithm provides more accurate and robust predictions in the high-confidence range. Among the individual categories, the scratches category performed the best, with significant precision improvements in the medium-to-low confidence range (0.3–0.6) and a smoother, more stable curve. The crazing category showed substantial precision improvements in the medium-to-high confidence range (0.6–0.9), reflecting enhanced stability. The patches category also exhibited noticeable improvements in the low-confidence range. Overall, the improved YOLOv9 algorithm achieves higher precision across all confidence ranges compared to the original algorithm, demonstrating stronger adaptability and stability across all categories.

## Conclusion

This study aims to improve the YOLOv9 model to enhance its performance in steel surface defect detection tasks. To address the shortcomings of the existing YOLOv9 model in small object detection, this paper proposes an improved YOLOv9 model by introducing the C3 module, DSConv convolution, the BiFPN module, and the DySample upsampling module. Detailed experimental validation of the proposed model was conducted. The main research findings are as follows:

Firstly, the introduction of the C3 module enables effective fusion of cross-stage features, allowing the transfer of critical semantic information across different scales. This enhances the network’s ability to recognize multi-scale defects. When processing steel surface images, the improved model can more accurately identify defects, effectively reducing missed and false detections.

Secondly, the incorporation of DSConv convolution optimizes the computational efficiency and feature extraction process of the model. By reducing computational complexity and refining feature processing, DSConv convolution maintains high-quality feature extraction while achieving high detection accuracy. Experimental results demonstrate that DSConv convolution significantly improves both speed and performance when handling high-resolution images.

Thirdly, the BiFPN module introduces a bidirectional feature fusion strategy based on the traditional Feature Pyramid Network (FPN), enhancing the flow of information between features at different scales. This module not only strengthens the fusion of high-resolution and low-resolution features but also avoids information loss. As a result, small defects on steel surfaces can be more precisely localized and classified. Experimental results show that the inclusion of the BiFPN module improves small object detection accuracy and effectively reduces missed detections.

Finally, the DySample upsampling operator dynamically adjusts the sampling points’ positions to further optimize the network’s ability to restore features in high-resolution regions. In the detection of small defects on steel surfaces, small objects often occupy small areas and have blurred details. Traditional upsampling methods may fail to accurately recover the detailed features of the targets. The DySample operator uses a dynamic sampling strategy to adaptively adjust the sampling regions based on the actual position and size of the defects, improving the precision of upsampling. This effectively avoids missed detections caused by insufficient feature recovery.

Although the proposed improved model demonstrates excellent performance across several key metrics, it still has some limitations. For instance, the inclusion of multiple new modules increases the model’s complexity, resulting in a slight extension of inference time (FPS). While the overall FPS of the improved model remains at an acceptable level, further optimization of the model’s speed performance may be required for industrial inspection scenarios where real-time requirements are more stringent.

## Data Availability

The datasets can be downloaded in https://github.com/sanyauChenCoder/gangcai_01.git.
